# Subcutaneous Dorsomedial Triangle of Forearm: Surgical Anatomy and Clinical Implication

**DOI:** 10.7759/cureus.41981

**Published:** 2023-07-16

**Authors:** Sheetal Kotgirwar, Sunita A Athavale, Rekha Lalwani, Manal M Khan, Ved Prakash Rao Cheruvu

**Affiliations:** 1 Anatomy, All India Institute of Medical Sciences, Bhopal, Bhopal, IND; 2 Plastic and Reconstructive Surgery, All India Institute of Medical Sciences, Bhopal, Bhopal, IND

**Keywords:** distal forearm, microsurgery, dorsal branch of ulnar nerve, dorsal branch of ulnar artery, arthroscopy

## Abstract

Background

The purpose of the study was to provide a practical landmark for localizing the dorsal branch of the ulnar artery and nerve, to approach for microsurgical flaps, for harvesting nerve grafts and also to avoid these nerves during insertion of wrist arthroscopy portals.

Material and methods

Forty adult cadaveric upper limbs (20 right and 20 left) were dissected for localizing the dorsal branches of the ulnar artery and nerve. The ramification patterns of the nerve were mapped. The wrist arthroscopy portals are located radial and ulnar to the tendon of extensor carpi ulnaris at the level of the wrist joint, and their designated names are '6R & 6U', respectively. The distance of branches of the nerve from the 6U and 6R portals for wrist arthroscopy was recorded.

Results

The present study has delineated a subcutaneous dorsomedial triangular area in the distal forearm. The construction of this triangle uses palpable landmarks, i.e. pisiform bone, styloid process and subcutaneous border of the ulna. The measure of the sides of the triangle uses proportion rather than absolute measurements and hence is person specific. The dorsal branches of the ulnar nerve and artery are consistently given off in the triangle's upper third and middle third, respectively.

Four branching patterns have been mapped, with one dominant pattern in 67.5% of limbs. In three-fourths of cases, one branch of the dorsal branch of the ulnar nerve consistently overlies the 6U portal and hence runs a higher risk of injury.

Conclusion

The study suggests more practical, accurate, reliable and consistent surface landmarks for the localization of the dorsal branch of the ulnar artery and nerve for reconstructive microsurgery for distal hand defects.

## Introduction

Soft tissue defects of the hand and wrist are a real challenge for plastic surgeons to preserve hand functions and allow early rehabilitation [1,2]. The anatomical knowledge of the dorsal branch of the ulnar nerve (DBUN) and ulnar artery (DBUA) plays a significant role in designing neurocutaneous flaps for reconstructive surgeries of the hand [3].

The dorsal cutaneous branch of the ulnar nerve is frequently injured during various surgeries on the ulnar side of the hand and during portal insertion during arthroscopy procedures and arthroscopic triangular fibrocartilage complex repair on the ulnar side [3-6].

The ulnar artery and nerve lie deep in the flexor carpi ulnaris muscle of the forearm. Standard anatomy texts describe the DBUA, a constant dorso-ulnar perforator, as 2-5 cm proximal to the pisiform bone. The DBUA is accompanied by DBUN, which arises 5 cm proximal to the wrist joint [7,8].

However, the precise location and relationship to other anatomical structures and landmarks have yet to be described, especially in the forearm and wrist joint proximity. The available studies also fail to give a comprehensive picture to the operating surgeons on the localization of arteries and nerves together.

Variations in the course and branches of DBUN can damage them during the insertion of wrist arthroscopy portals. The study of DBUN can provide a safe zone for portal insertion during the procedure. It also can aid in locating the nerve for repair or graft. The present study intends to describe the DBUN in the forearm and its proximity to arthroscopy portals of the wrist joint.

In addition, to provide data regarding DBUA to aid plastic surgeons in longer vascular pedicles for designing dorsal cutaneous flaps for better reconstructive microsurgical procedures of hand defects.

## Materials and methods

Forty formalin-fixed embalmed upper limbs of adult cadavers were included in the study. Limbs with evidence of hand deformities, hand trauma and hand surgeries were excluded.

**Figure 1:**
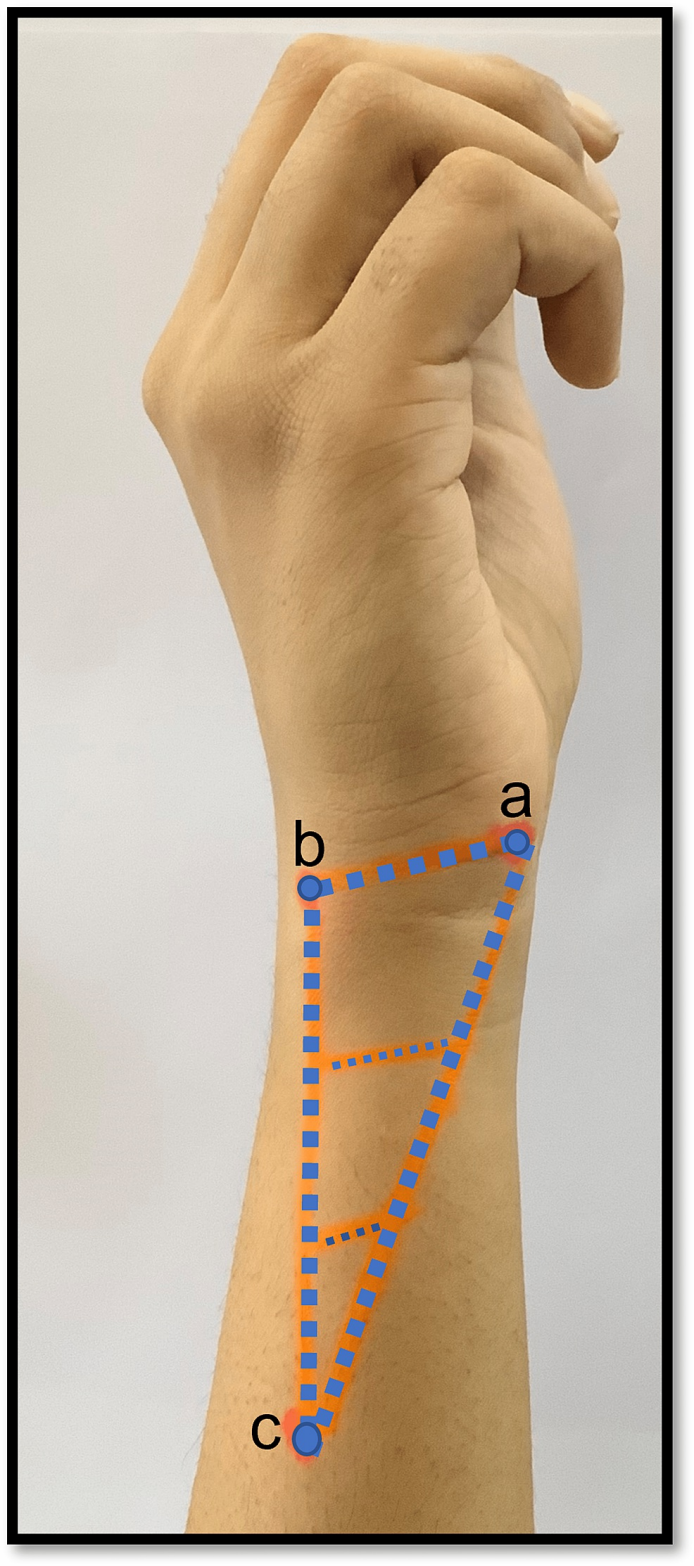
Showing the surface marking of the subcutaneous dorsomedial triangle. a=proximal border of pisiform bone; b=tip of styloid process of ulna; c=apex of the triangle.

To describe the precise location and course of the dorsal branch of the ulnar nerve and artery with palpable landmarks, a triangular area in the distal forearm on the medial aspect was marked by joining the points as follows: 'a'- Proximal border of pisiform bone, 'b'-Tip of the styloid process of the ulna. The triangle's base was marked by joining points 'a' and 'b'. The distance between these points was measured with the help of thread and scale. A line was drawn extending from point 'b' along the posterior subcutaneous border of the ulna, measuring twice the distance between points 'a' and 'b'. A point 'c' was marked at the termination of this line on the subcutaneous border of the ulna. The triangle was completed by a line joining points 'a' and 'c'. This resulted in the demarcation of a triangular area located on the dorsomedial aspect of the lower third of the forearm, which was subcutaneous. The triangle was subdivided into three approximately equal parts by drawing lines parallel to the base (Figure 1).

The skin over this triangle was reflected. Skirting the ulnar border of the triangle, careful dissection of subcutaneous tissue was carried out to locate dorsal branches of ulnar nerve and artery.

The distance of emergence of the DBUA, from the ulnar artery, from the proximal border of the pisiform bone was measured with the help of digital vernier calipers.

The subcutaneous tissue was carefully cleaned to localize and map the nerve within the triangle. 6U and 6R portal of wrist arthroscopy are located just ulnar and radial to extensor carpi ulnaris tendon (ECU) at the level of the wrist joint. To ascertain the proximity of branches of DBUN, their closest distance from the wrist arthroscopy portals 6U and 6R was also measured.

## Results

Upon reflection of the skin over the triangular area, as defined, it was observed that the ventral (ulnar) margin closely abutted the medial border of flexor carpi ulnaris (FCU). In contrast, the dorsal (radial) margin ran closely along the medial border of the ECU.

**Figure 2:**
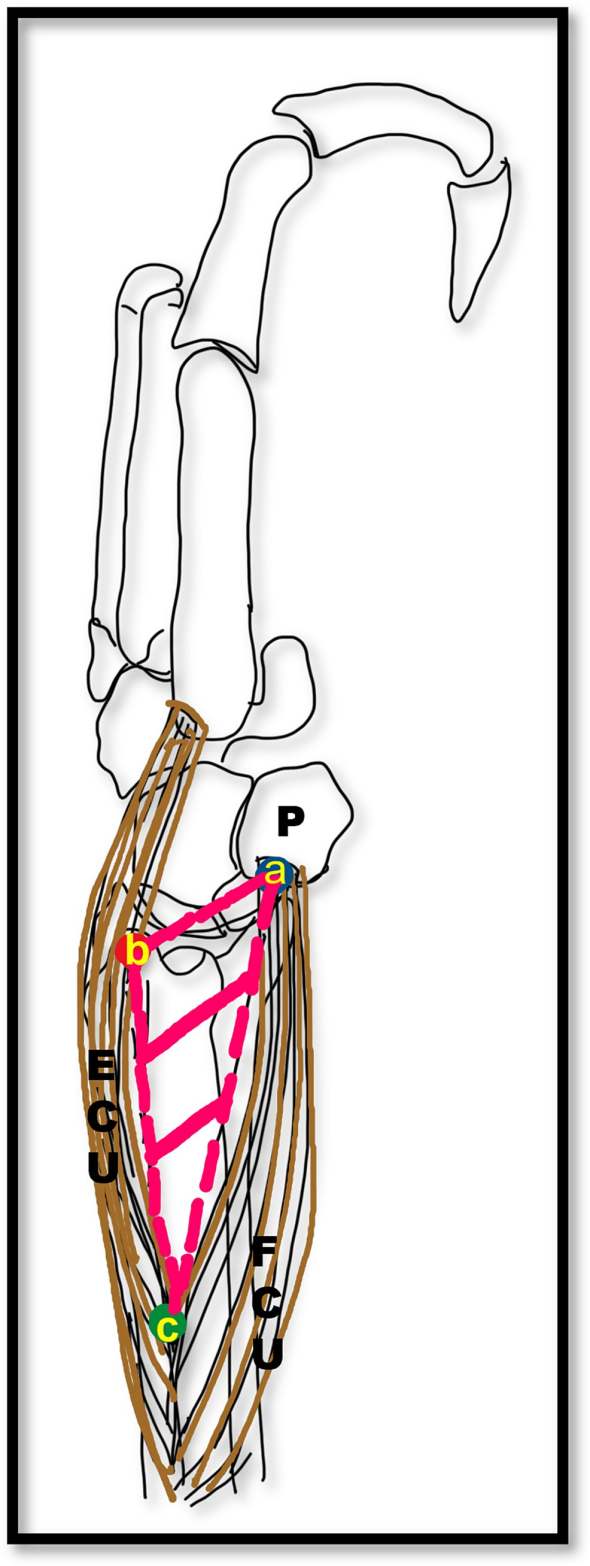
Line diagram showing the boundaries of the subcutaneous dorsomedial triangle. Line diagram showing the boundaries of the subcutaneous dorsomedial triangle. a=proximal border of pisiform bone; b=tip of styloid process; c=apex of the triangle; ECU=extensor carpi ulnaris; FCU=flexor carpi ulnaris; P=pisiform bone.

**Figure 3:**
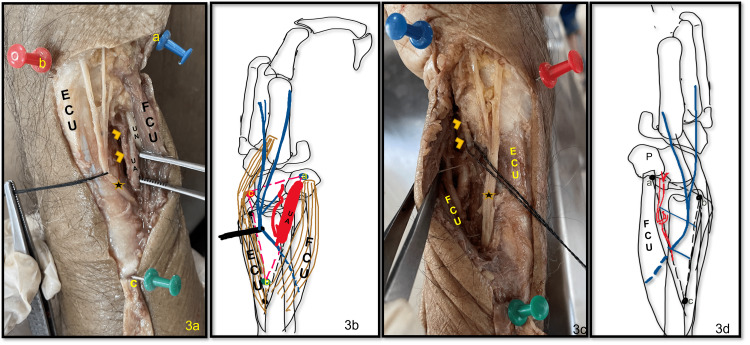
a-d showing the contents of the subcutaneous dorsomedial triangle. 3a shows the DBUN ('star') running in the triangle dividing into three branches just proximal to the styloid process, and DBUA ('arrowheads') is seen. 3b line diagram showing the same. 3c shows the DBUN dividing into two branches. 3d line diagram of the photograph in 3c. a=proximal border of pisiform bone; b=tip of styloid process; c=apex of the triangle; ECU=extensor carpi ulnaris; FCU=flexor carpi ulnaris; DB=dorsal branch; UA=ulnar artery; UN=ulnar nerve.

At the apex of the triangle, both the muscles were close to each other, owing to their origin from the proximal subcutaneous border of the ulna. The floor of the triangle was formed by the lower part of the shaft of the ulna (Figure 2). This subcutaneous triangle contained the DBUA and DBUN (Figure 3A-3D). This triangle was named as subcutaneous dorsomedial (SDM) triangle of the forearm.

The DBUA was given out about 3.48 ± 0.98 centimeters proximal to the proximal border of the pisiform bone. The artery coursed medially to emerge in this triangle along the medial border of FCU (Figure 3A-3D). It pierced the deep fascia as soon as it entered the triangle and divided into ascending and descending branches. The site of the emergence of the DBUA was located in the lower part of the middle third of the triangle.

**Figure 4:**
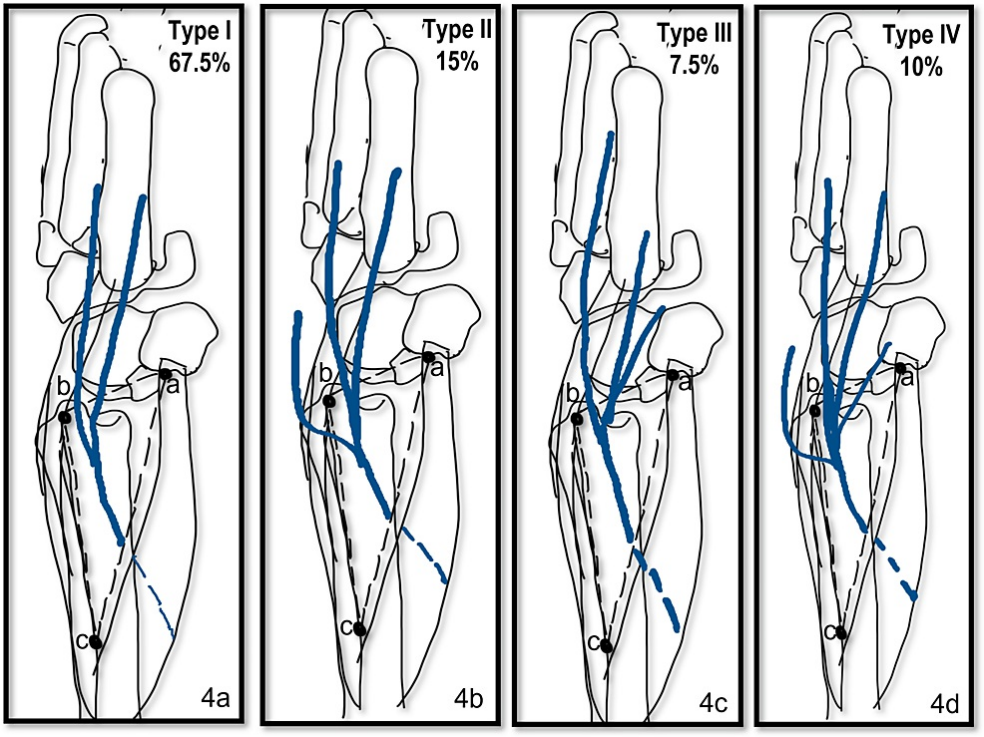
(a-d) shows the line diagram showing the varied branching pattern of DBUN within the triangle. a=proximal border of pisiform bone; b=tip of styloid process; c=apex of the triangle; DBUN=dorsal branch of the ulnar nerve

The DBUN was given out deep to the FCU and emerged into the triangle along its medial border in the lower part of the upper third of the triangle. It traversed the triangle obliquely and in the middle one-third of the triangle and reached close to the medial margin of ECU. In the distal third of the triangle, the nerve is divided into digital branches for supply to little and ring finger. Four different types of patterns of branching of DBUN were observed as shown in Figure 4A-4D.

Type I was the most common pattern observed in 27 of 40 - 13 right and 14 left (67.5 %) limbs. The division was found proximal to the styloid process in 23/40 (12 right and 11 left) and distal to it in four (one right and three left) limbs. Two digital branches were given out, the medial one coursed straight towards the ulnar side of the little finger, and the lateral ran close to the ECU tendon, crossed it at a variable distance distal to the styloid process, and proceeded towards the fourth interdigital cleft. This branch lies on or very close to the 6U portal for wrist arthroscopy (Figure 3C, 3D).

**Figure 5:**
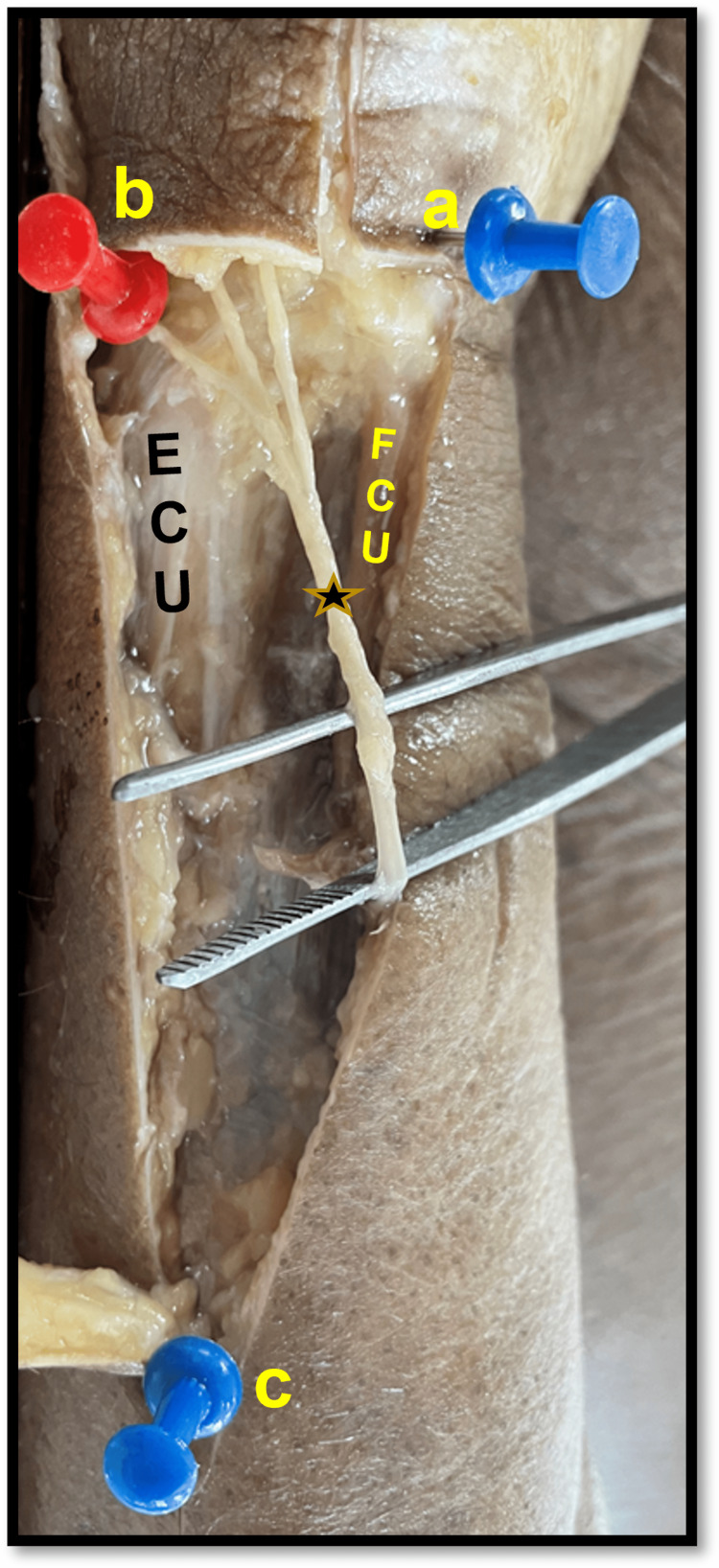
Showing DBUN ('star') dividing into three branches. The dorsal branch (running under the red pin) overlies the 6R portal. a=proximal border of pisiform bone; b=tip of styloid process; c=apex of the triangle; ECU=extensor carpi ulnaris; FCU=flexor carpi ulnaris; P=pisiform bone; DBUN=dorsal branch of the ulnar nerve

In type II, besides the two digital branches, one extra branch was given out, which crossed the ECU tendon proximal to the styloid process and courses toward the third interdigital cleft. This branch was seen in six of 40 (four right and two left; 15%) limbs and was found to lie very close to the 6R of the wrist arthroscopy portal (Figure 5).

In type III, one extra branch was given out, which coursed ventrally towards the palm and was seen in three (one right and two left; 7.5%) limbs (Figure 3A). This branch coursed towards the wrist capsule.

In type IV, this pattern combined variants type II and III occurring together. This was observed in four limbs (two each on right and left; 10%).

It was observed that the digital branches of DBUN to the fourth interdigital cleft coursed very close to the 6U portal, and almost three-fourths of limbs were lying at the site of the 6U portal.

The branch of DBUN was close to the 6R portal in type II and IV variants, when an additional branch was observed very close to the portal site. The nerve was seen on the 6R portal in eight limbs (four right and four left). Table 1 shows the morphometric variables of DBUA and DBUN.

**Table 1:** Shows the morphometric variables of DBUA and DBUN DBUA=dorsal branch of the ulnar artery; DBUN=dorsal branch of the ulnar nerve

Distance of	Mean	SD	Minimum	Maximum
DBUA from the proximal border of Pisiform (mm)	34.80	9.87	3.40	6.90
DBUN from 6U (mm)	1.95	20.16	0.00	0.00
DBUN from 6R (mm)	9.74	63.88	11.59	21.42

## Discussion

Dorsal ulnar artery perforator flaps are preferred for reconstructing digital defects owing to several advantages, viz.: the presence of a constant perforator, thin, pliable, and hairless skin in the area and can be harvested under regional anesthesia [9-11]. Malkoc et al. [12] have reported the origin of DBUA 4.1 cm (ranging 3.2-5.5 cm) proximal to pisiform in 10 cadaveric specimens studied. DBUA was seen to arise at a distance of 3.4 cm proximal to the pisiform; however large standard deviation suggests wide variability in the origin of the dorsal branch of the ulnar artery. Juhui et al. [10] have also reported that wide variability in the origin of DBUA (ranging from 2 cm to 6 cm) was observed in surgical cases operated for hand reconstruction surgeries.

Arthroscopic repair of the triangular fibrocartilagenous disc is increasingly becoming common and occasional cases of injury to DBUN have also been reported [13]. Anatomical studies describing DBUN have primarily used the ulnar styloid process as a landmark to describe the location and course [4,5,14]. Uerpairokit et al. [14] studied the pattern in 44 limbs and related and classified the nerve according to its crossing a vertical axis. Goto et al. [5] described the nerve to divide proximal to the ulnar styloid in more than two-thirds of the limbs studied. Similarly, Tindall et al. [4] described the nerve crossing an imaginary line joining the ulnar styloid process and the first web space on the dorsum of the hand. Cavosoglu et al. [3] described the origin and point of piercing deep fascia with respect to the medial epicondyle. They also observed that the DBUN was consistently divided into two branches that supplied the digits. These anatomical studies, however, do not mention the proximity of the nerve to the wrist arthroscopy portals towards the ulnar aspect. They also fail to describe the course of the nerve in the area used for neurocutaneous flaps. The present study described the course of DBUN within the triangle and documented four nerve branching patterns. Similar to Cavosoglu et al. [3] the present study observed a predominant pattern of two branches being given out proximal to the styloid process in the lower third of the triangle; however, the authors have additionally documented variant patterns.

As observed in the present study, one of the branches of DBUN consistently lays on or very close to the 6U portal of wrist arthroscopy in three fourth cases, and another branch was close to the 6R portal in one-fourth of the cases. These findings call for exercising caution while using this portal for arthroscopy.

Poublon et al. [6], in their study on 20 cadaveric limbs, state that no safe zone could be identified for the 6R portal however have not commented on the 6U portal; however, our observations reveal that the 6U portal is more likely to be in contact with the nerve as compared to 6R portal.

The present study used palpable landmarks to describe the comprehensive anatomy of a subcutaneous dorsomedial (SDM) triangle of the forearm. Knowledge of this subcutaneous triangle has the following advantage in understanding the comprehensive anatomy of the dorsoulnar region of the hand: a) It demarcates an area with thin, pliable, and hairless skin. The location of DBUA and DBUN can be described together. The division of the SDM triangle into three parts helps localize the structures from the surface. The construction of the SDM triangle uses proportions rather than absolute dimensions and hence is person specific and easy to use during surgeries as it does not involve complicated or precise measurements.

Limitation

A larger sample size could have led to the description of more variant patterns.

## Conclusions

The study describes a subcutaneous triangular area with DBUA and DBUN as its contents which shall help in harvesting neurocutaneous flaps for distal hand defects.

6U and 6R portals of wrist arthroscopy were very close to the branch of DBUN in three-fourths and one-fourth of limbs, respectively. This calls for caution during inserting arthroscopy portals.
